# The proinflammatory LTB_4_/BLT1 signal axis confers resistance to TGF-β1-induced growth inhibition by targeting Smad3 linker region

**DOI:** 10.18632/oncotarget.6146

**Published:** 2015-10-19

**Authors:** Woo-Kwang Jeon, Jiyeon Choi, Seong Ji Park, Eun Ji Jo, Young K. Lee, Seunghwan Lim, Jae-Hong Kim, John J. Letterio, Fang Liu, Seong-Jin Kim, Byung-Chul Kim

**Affiliations:** ^1^ Department of Biochemistry, College of Natural Sciences, Kangwon National University, Chuncheon, Republic of Korea; ^2^ Department of Statistics, College of Natural Sciences, Kangwon National University, Chuncheon, Republic of Korea; ^3^ Department of Pediatrics, Case Comprehensive Cancer Center, Case Western Reserve University, Cleveland, Ohio, USA; ^4^ College of Life Sciences and Biotechnology, Korea University, Seoul, Korea; ^5^ Center for Advanced Biotechnology and Medicine, Susan Lehman Cullman Laboratory for Cancer Research, Ernest Mario School of Pharmacy, Rutgers Cancer Institute of New Jersey, Rutgers, The State University of New Jersey, Piscataway, NJ, USA; ^6^ CHA Cancer Institute and Department of Biomedical Science, College of Life Science, CHA University, Seongnam City, Republic of Korea

**Keywords:** LTB_4_, BLT1, Smad3 linker region phosphorylation, TGF-β1 resistance, cancer cell growth

## Abstract

Leukotriene B4 (LTB_4_) is a potent pro-inflammatory eicosanoid that is derived from arachidonic acid, and its signaling is known to have a tumor-promoting role in several cancer types. In this study, we investigated whether enhanced LTB_4_ signaling confers resistance to the cytostatic transforming growth factor-β1 (TGF-β1) response. We found that LTB_4_ pretreatment or ectopic expression of BLT1, a high affinity LTB_4_ receptor, fully abrogated TGF-β1-induced cell cycle arrest and expression of p15^INK4B^ and p27^KIP1^. Mechanism study revealed that LTB_4_-mediated suppression of TGF-β1-induced Smad3 activation and growth inhibition was due to enhanced phosphorylation of Smad3 linker region (pSmad3L) through activation of BLT1-NAD(P)H oxidase (NOX)-reactive oxygen species (ROS)-epidermal growth factor receptor (EGFR)-phosphatidylinositol 3-kinase (PI3-K)-extracellular signal-activated kinase1/2 (ERK1/2)-linked signaling cascade. Furthermore, the LTB_4_/BLT1 signaling pathway leading to pSmad3L was constitutively activated in breast cancer cells and was correlated with TGF-β1-resistant growth of the cells *in vitro* and *in vivo*. In human breast cancer tissues, the expression level of pSmad3L (Thr179) had a positive correlation with BLT1 expression. Collectively, our data demonstrate for the first time that the induction of pSmad3L through BLT1-NOX-ROS-EGFR-PI3K-ERK1/2 signaling pathway is a key mechanism by which LTB_4_ blocks the anti-proliferative responses of TGF-β1, providing a novel mechanistic insight into the connection between enhanced inflammatory signal and cancer cell growth.

## INTRODUCTION

Chronic inflammation is a risk factor for cancer. Inflammation mediators, including cytokines and chemokines act to create a favorable microenvironment for the progression of tumor, and inflammatory signaling pathways are activated in many types of cancer [1]. However, the molecular signaling mechanisms underlying the contribution of inflammatory signal to cancer development remain to be elucidated.

Leukotriene B_4_ (LTB_4_), an oxidized fatty acid derived from a multi-step 5-lipoxygenase (5-LO) pathway in arachidonic acid metabolism, is a well-known chemotactic agent for recruitment to, activation, and survival of phagocytes at the site of tissue injury [2, 3]. Overexpression of LTB_4_ is frequently observed in the airways of asthma patients and its levels are correlated with the severity of asthma [4], indicating its importance in inflammatory disease processes. Recently, mounting evidences suggest that 5-LO metabolites are also linked to the pathogenesis of a variety of human cancers, including colon, liver, and pancreatic cancers. For example, the blockade of LTB_4_-extracellular signal-regulated kinase 1/2 (ERK1/2) pathway suppresses the proliferation and survival of colon cancer cells [5]. LTB_4_ also is a potential mediator of oncogenic hepatitis B virus X-induced proliferation of hepatoma cells [6]. Celecobix, a cycloxygenase-2 inhibitor, exerts its anti-cancer effect primarily via down-regulating LTB_4_ production in colon cancer cells [7]. In addition, two LTB_4_ receptor subtypes, BLT1 and BLT2, are overexpressed in various human cancers [8, 9]. LY293111, an antagonist of BLT1, induces apoptosis in human pancreatic cancer and lymphoma cells and reduces the growth of tumor xenografts [10, 11]. Although these evidences clearly suggest that LTB_4_/BLT axis may play an important role in the progression of human cancer by increasing proliferation and survival, the molecular mechanisms remain to be elucidated.

Transforming growth factor-β1 (TGF-β1) is an important cytokine that modulates diverse cellular functions [12]. The most well defined TGF-β1 response is cell growth inhibition. TGF-β1 causes cell cycle arrest at the G_1_ phase by suppressing cyclin-dependent kinase activity through the induction of p15^INK4B^ and p21^WAF1^ [13, 14]. Another key event in the anti-proliferative TGF-β1 action is down-regulation of growth promoting factors such as c-Myc and Id1, and those responses are lost in certain types of tumor cells [15–17]. Therefore, maintenance of the cytostatic function of TGF-β1 is important for prevention of early-stage cancer development [17].

TGF-β1 transmits signals from the cell surface to the nucleus by activating a signaling cascade that involves Smad proteins through a hetero-oligomerization of type I and type II transmembrane TGF-β receptors (TβRI and TβRII) [17]. Smad3, one component of the Smad signaling pathway, plays a central role in mediating TGF-β1 growth inhibitory signal from receptors to the nucleus. For example, TGF-β1-stimulated Smad3 binds to a Smad-E2F site on the c-myc promoter together with cofactors p107 and E2F4/5, resulting in c-myc repression [17, 18], whereas overexpressed Smad3 induces transcription of the CDK inhibitor p21^WAF1^ through functional cooperation with the transcription factor Sp1, driving G_1_ cell cycle arrest [19, 20].

The Smad3 protein is composed of highly conserved N-terminal MH1 domain (Mad-homology-1) responsible for DNA binding and C-terminal MH2 domain (Mad-homology-2) responsible for transactivation, which are connected by a proline-rich linker region. Accumulating evidences indicate that the linker region of Smad3 contains phosphorylation sites for several classes of protein kinases and serves an important function in regulating its transcriptional activity under physiologic and pathologic conditions. For example, mitogenic signal-activated cyclin-dependent kinase (CDK) or extracellular signal-regulated kinase 1/2 (ERK1/2) causes phosphorylation of Smad3 at the linker region, resulting in suppression of TGF-β1-mediated Smad3 transcriptional activation and growth inhibition [21, 22]. In contrast, linker region phosphorylated Smad3 functions as a stimulator of cell growth and invasion in oncogenic Ras-transformed epithelial cells [23]. Based on these clues, the Smad3 linker region phosphorylation (pSmad3L) closely associates with cancer progression, although the detailed cellular signaling mechanisms responsible for the phosphorylation and its biological significance have not been fully understood.

In this study, we showed that LTB_4_ negatively regulates the anti-proliferative TGF-β1 signaling by targeting Smad3 linker region. In mammary epithelial cells, LTB_4_ strongly induced phosphorylation of Smad3 at the linker region via a BLT1-NOX-ROS-EGFR-PI3K-ERK1/2-linked signaling cascade, resulting in decreased transcriptional activity of Smad3 and eventually impaired growth inhibition response to TGF-β1. We also found that the LTB_4_ signaling pathway leading to pSmad3L was markedly activated in MDA-MB231 human breast cancer cells and was correlated with TGF-β1-resistant growth of the cells *in vitro* and *in vivo*. Furthermore, the level of pSmad3L was higher in human breast cancer tissues compared with that of normal counterparts and positively correlated with BLT1 expression. These findings provide an important mechanistic insight into the connection between inflammatory LTB_4_ signal and cancer cell growth.

## RESULTS

### LTB_4_/BLT1 axis inhibits TGF-β1-induced cell cycle arrest

**Figure 1:**
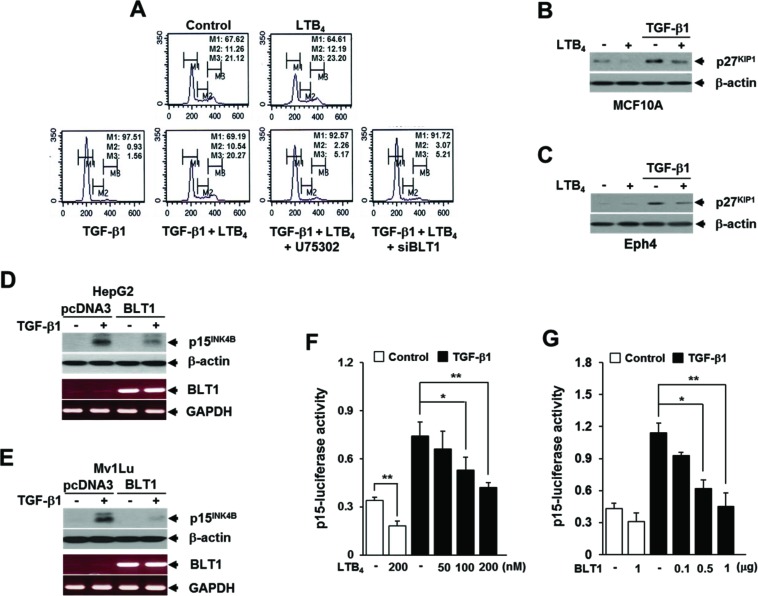
LTB_4_/BLT1 axis inhibits TGF-β1-induced G_1_ arrest and expression of p27^KIP1^ and p15^INK4B^ **A.** Upper panels: MCF10A cells were incubated with or without 5 ng/ml of TGF-β1 for 24 h in the absence or presence of 100 nM of LTB_4_. Lower panels: MCF10A cells pretreated with 10 μM of U75302 or transfected with BLT1 siRNA were incubated with or without 5 ng/ml of TGF-μ1 for 24 h in the absence or presence of 100 nM of LTB_4_. Cells were then stained with propodium iodide and subjected to FACS analysis. The percentage of cells in G1 was designated as M1, S as M2, and G2/M as M3. **B.** MCF10A and **C.** Eph4 cells were pretreated with EtOH (vehicle) or 100 nM of LTB_4_ for 30 min and then stimulated with 5 ng/ml of TGF-β1 for 24 h. The cell lysates were analyzed by immunoblot for p27^KIP1^ level. Stable **D.** HepG2 and **E.** Mv1Lu cell lines that expressing the control vector (pcDNA3) or BLT1 were stimulated with 5 ng/ml of TGF-β1 for 24 h. The cell lysates were analyzed by immunoblot for p27^KIP1^protein level and by semiquantitative RT-PCR for BLT1 mRNA level. β-actin and GAPDH levels were monitored as controls. **F.** HepG2 cells transfected with p15^INK4B^-luciferase reporter plasmid were pretreated with LTB_4_ at the indicated concentrations for 30 min and then stimulated with 5 ng/ml of TGF-β1 for 24 h. **G.** HepG2 cells co-transfected with p15^INK4B^-luciferase reporter plasmid together with the indicated amount of BLT1 plasmid were stimulated with 5 ng/ml of TGF-β1 for 24 h. Luciferase activities were normalized on the basis of β-galactosidase expression to adjust for variation in transfection efficiency. All quantitative data are shown as the mean ± SD of three independent experiments. *p < 0.05, **p < 0.01.

Since LTB_4_ and its cognate receptors are implicated in promotion of cancer cell growth [8, 9], we investigated whether LTB_4_ signal would influence the antiproliferative effect of TGF-β1. Cell cycle profiling by FACS analysis showed that pretreatment of MCF10A cells with LTB_4_ completely inhibits TGF-β1-induced G_1_ cell cycle arrest, but the effect is greatly diminished by either pretreatment with BLT1 antagonist U75302 or transfection with BLT1 small interference (si) RNA (Figure 1A). Down-regulation of endogenous BLT1 protein expression by siRNA BLT1 was confirmed by immunoblotting (Supplementary Figure S1). In agreement with these results, TGF-β1-induced p27^KIP1^ protein expression was significantly inhibited in MCF10A (Figure 1B) and Eph4 (Figure 1C) mammary epithelial cells that pretreated with LTB_4_. Similarly, TGF-β1-induced p15^INK4B^ protein expression was strongly attenuated in both HepG2-BLT1 (Figure 1D) and Mv1Lu-BLT1 (Figure 1E) stable cell lines that overexpress BLT1 compared to that of their control pcDNA3 vector cell lines. Consistently, TGF-β1-induced p15^INK4B^-Luc reporter gene expression was inhibited by LTB_4_ pretreatment (Figure 1F) or ectopic expression of BLT1 (Figure 1G) in a dose-dependent manner in HepG2 cells. These results strongly suggest that LTB_4_/BLT1 axis suppresses the anti-proliferative function of TGF-β1.

### LTB_4_/BLT1 axis inhibits TGF-β1-induced Smad3 activation and G_1_ arrest through increasing Smad3 linker region phosphorylation

**Figure 2:**
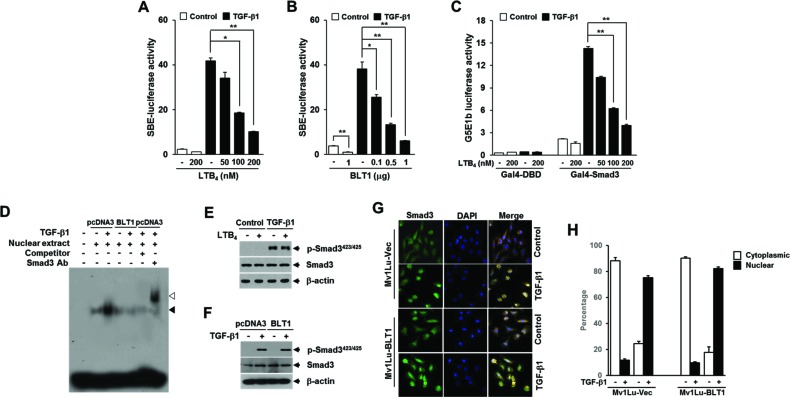
LTB_4_/BLT1 axis inhibits TGF-β1-induced Smad3 transactivation without affecting Smad3 C-terminal phosphorylation and its translocation into the nucleus **A.** HepG2 cells transfected with Smad-binding element (SBE)-luciferase reporter plasmid were pretreated with LTB_4_ at the indicated concentrations for 30 min and then stimulated with 5 ng/ml of TGF-β1 for 24 h. **B.** HepG2 cells co-transfected with SBE-luciferase reporter plasmid together with the indicated amounts of BLT1 plasmid were incubated with or without 5 ng/ml of TGF-β1 for 24 h. **C.** HepG2 cells co-transfected with G5E1b-luciferase plasmid together with Gal4-DBD or Gal4-Smad3 plasmid were pretreated with LTB_4_ at the indicated concentrations for 30 min and then stimulated with 5 ng/ml of TGF-β1 for 24 h. Luciferase activities were normalized as in Fig. 1 **F.** and **G.**. All quantitative data are shown as the mean ± SD of three independent experiments. *p < 0.05, **p < 0.01. **D.** Stable Mv1Lu-pcDNA3 and Mv1Lu-BLT1 cell lines were incubated without or with 5 ng/ml of TGF-β1 for 2 h, and nuclear extracts were subjected to gel shift assay using probe containing four copies of SBE. Black arrow indicates the position of the Smad3-DNA complex. The supershifted band (white arrow) was observed upon addition of the Smad3 antibody to the binding reaction. **E.** MCF10A cells pretreated with EtOH (vehicle) or 100 nM of LTB_4_ for 30 min were stimulated with 5 ng/ml of TGF-β1 for 30 min. The protein levels of Smad3 and its phosphorylation were analyzed by immunoblot with Smad3 and phospho-Smad3 (Ser423/425) antibodies. β-actin levels were monitored as a control. **F.** Mv1Lu-pcDNA3 and Mv1Lu-BLT1 cell lines were treated without or with TGF-β1 and then analyzed for Smad3 and phospho-Smad3 (Ser423/425) levels as in **E.**. **G.** Stable Mv1Lu-pcDNA3 and Mv1Lu-BLT1 cell lines were treated with or without 5 ng/ml of TGF-β1 for 30 min. Cells were fixed with 3.5% paraformaldehyde, permeabilized, and immunostained for Smad3 (Alexa 488; green). The nuclei were stained with 4,6-diamidino-2-phenylindole (DAPI; blue). The merger of Alexa 488 and DAPI is shown in the right panel. Magnification, 40x. The images presented here are representative of multiple fields from three independent experiments. **H.** Histogram showing the results of three independent experiments; random fields were selected and the staining pattern of each cell line within the field was scored visually. 250 cells were scored for each cell line. Plotted is the percentage of cells in each category ±SD; there is no significant difference between the percent nuclear for the two cell lines (*p* < 0.05).

We next explored the mechanisms by which LTB_4_ inhibits TGF-β1-induced cell cycle arrest. Because Smad3 is well known to have an essential role in mediating TGF-β growth inhibitory signal from the receptors to the nucleus, we examined the influence of LTB_4_/BLT1 axis on TGF-β1-stimulated Smad3 transcriptional activity. To do this, we used the artificial SBE_4_-Luc reporter, which comprises four tandem repeats of Smad-binding elements (SBEs) and measures a Smad3/4-specific response [29]. As shown in Figure 2A and 2B, pretreatment with LTB_4_ or ectopic expression of BLT1 resulted in a dose-dependent inhibition of TGF-β1-induced SBE_4_-Luc reporter gene expression in HepG2 cells. In addition, LTB_4_ suppressed TGF-β1-stimulated transcriptional activity of GAL4-Smad3 fusion protein in a concentration-dependent manner (Figure 2C). Consistent with these results, electrophoretic mobility-shift assay revealed that the increased binding affinity of Smad3 to SBE in response to TGF-β1 is markedly diminished in HepG2-BLT1 cells compared with HepG2-pcDNA3 control cells (Figure 2D). However, in Mv1Lu cells pretreated with LTB_4_, no difference on Smad3 C-terminus phosphorylation was seen with TGF-β1 treatment compared with LTB_4_-untreated cells (Figure 2E). Similarly, the C-terminus phosphorylation of Smad3 in Mv1Lu-BLT1 cells was comparable with that of control Mv1Lu-pcDNA3 cells after TGF-β1 treatment (Figure 2F). We also found that TGF-β1 treatment causes the nuclear accumulation of Smad3 in Mv1Lu-BLT1 cells without significant difference to that seen in control Mv1Lu-pcDNA3 cells (Figure 2G and 2H). These results indicate that LTB_4_-BLT1 axis suppresses the transcriptional activity of Smad3 without affecting its C-terminus phosphorylation and nuclear accumulation under TGF-β1 stimulation.

**Figure 3:**
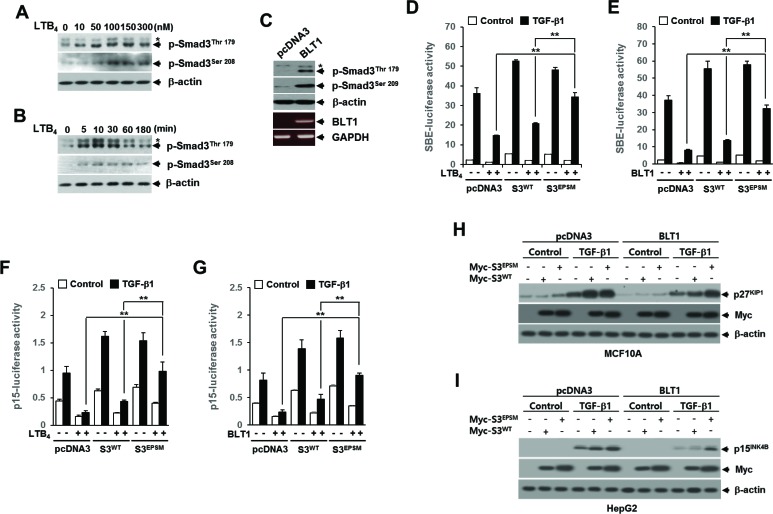
The LTB_4_/BLT1 axis inhibits TGF-β1-induced Smad3 transactivation and cell cycle arrest through increasing Smad3 linker region phosphorylation MCF10A cells were incubated **A.** with the indicated concentrations of LTB_4_ for 30 min, or **B.** with 100 nM of LTB_4_ for the indicated times. The cell lysates were analyzed by immunoblot with specific phosphopeptide antibodies against the phosphorylated Thr179 and Ser208 in the Smad3 linker region. **C.** Whole cell lysates from stable Mv1Lu-pcDNA3 and Mv1Lu-BLT1 cell lines were analyzed by immunoblot as in **A.**. Asterisk represents a phospho-Smad2 (Thr220). The BLT1 mRNA level was analyzed by semiquantitative RT-PCR. β-actin and GAPDH levels were monitored as controls. HepG2 cells were co-transfected with **D.** SBE-luciferase reporter plasmid or **F.** p15^INK4B^-luciferase reporter plasmid together with pcDNA3, Smad3^WT^, or Smad3^EPSM^ plasmid. At 24 h after transfection, cells were treated with EtOH (vehicle) or 100 nM of LTB_4_ for 30 min, and then stimulated with 5 ng/ml of TGF-β1 for 24 h. **E.** and **G.** Cells were transfected and then stimulated with TGF-β1 as in **D.** and **F.** excepting co-transfection with BLT1 plasmid instead of LTB_4_ treatment. All quantitative data are shown as the mean ± SD of three independent experiments. **p < 0.01. Luciferase activities were normalized on the basis of β-galactosidase expression to adjust for variation in transfection efficiency. **H.** Stable MCF10A-pcDNA3 or MCF10A-BLT1 cell lines infected with mock, Myc-Smad3^EPSM^ or Myc-Smad3^WT^ lentiviruses were stimulated with 5 ng/ml of TGF-β1 for 24 h. **I.** Stable Mv1Lu-pcDNA3 and Mv1Lu-BLT1 cell lines infected with mock, Myc-Smad3^EPSM^ or Myc-Smad3^WT^ lentiviruses were stimulated with 5 ng/ml of TGF-β1 for 24 h. The cell lysates were analyzed by immunoblot with a specific antibody against p27^KIP1^, p15^INK4B^, and Myc, respectively. β-actin levels were monitored as a control.

There is a growing body of evidences pointing to an important role of Smad3 linker region phosphorylation (pSmad3L) in the regulation of Smad3 function under physiologic and pathologic conditions [21, 22] So, we examined whether LTB_4_/BLT1 axis affects the pSmad3L. Western blot analysis showed that LTB_4_ increases the amounts of Smad3 phosphorylated at two serine and threonine residues (Thr179 and Ser208) in the linker region in a concentration-dependent manner (Figure 3A). Concentrations of LTB_4_ as low as 100 nM were capable of inducing maximal phosphorylation of Smad3 at Thr179 or Ser208. Using 100 nM LTB_4_, induction reached a maximum after 10 minutes of treatment with LTB_4_ (Figure 3B). Consistent with this result, the Smad3 phosphorylation at Thr179 or Ser208 was markedly enhanced in Mv1Lu-BLT1 cells compared with control Mv1Lu-pcDNA3cells (Figure 3C), raising a possibility that Smad3 linker region can be a potential target of LTB_4_/BLT1 axis to blocks the anti-proliferative TGF-β1 signal.

To determine whether pSmad3L contributes to the LTB_4_-mediated inhibition of TGF-β1-induced Smad3 transcriptional activity and G_1_ cell cycle arrest, we used Smad3 mutated at all four of the linker region phosphorylation sites, including Thr179, Ser204, Ser208, and Ser213, called Smad3^EPSM^ [30]. In HepG2 cells, the LTB_4_-, and BLT1-mediated inhibitions of TGF-β1-induced SBE_4_-Luc (Figure 3D and 3E) or p15^INK4B^-Luc (Figure 3F and 3G) reporter gene expression were significantly reduced by co-transfection with Smad3^EPSM^, but not by co-transfection with Smad3^WT^. Similarly, western blot analysis showed that BLT1-mediated suppressions of TGF-β1-induced p27^KIP1^ (Figure 3H) and p15^INK4B^ (Figure 3I) expression were significantly attenuated by ectopic expression of Smad3^EPSM^, but not by ectopic expression of Smad3^WT^. Together, these results suggest that the pSmad3L is involved in the LTB_4_- and BLT1-mediated suppression of TGF-β1-induced Smad3 transcriptional activity and G_1_ cell cycle arrest.

### ROS production through the BLT1-NOX-linked cascade is required for the LTB_4_-mediated induction of pSmad3L and inhibition of TGF-β1-induced cell cycle arrest

**Figure 4:**
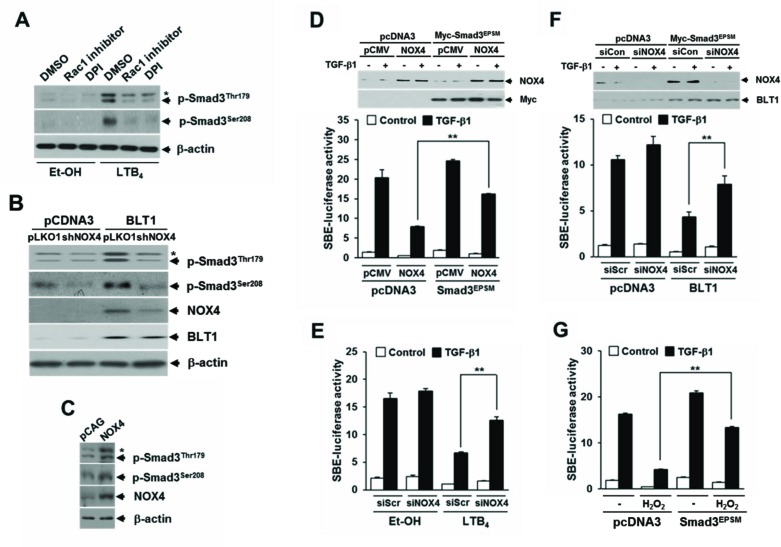
NOX is required for LTB_4_/BLT1-mediated induction of Smad3 linker region phosphorylation and inhibition of TGF-β1-stimulated SBE-Luc reporter activity **A.** MCF10A cells were pretreated with DMSO, 5 μM of Rac1 inhibitor, or 2.5 μM of DPI for 30 min, and then stimulated with EtOH (vehicle) or 100 nM of LTB_4_ for 30 min. **B.** Stable HepG2-pcDNA3 and HepG2-BLT1 cell lines were infected with pLOK1 vector or shNOX4 lentiviruses. **C.** MCF10A cells were infected with mock or NOX4 lentiviruse. Whole cell extracts were analyzed by immunoblot with a specific antibody against NOX4, phospho-Smad3 (Thr179), and phospho-Smad3 (Ser208), respectively. β-actin levels were monitored as a control. Asterisk represents a phospho-Smad2 (Thr220). **D.** HepG2 cells co-transfected with SBE-luciferase reporter plasmid together with control (scrambled, Scr) or NOX4 siRNAs were treated with EtOH (vehicle) or 100 nM of LTB_4_ for 30 min and then stimulated with 5 ng/ml of TGF-β1 for 24 h. Upper panel show the immunoblotting result of plasmid transfection. Lower panel show the measured luciferase activity. **E.** HepG2 cells were co-transfected with SBE-luciferase reporter plasmid and either pcDNA3 or BLT1 plasmid together with control (scrambled, Scr) or NOX4 siRNAs and then stimulated with 5 ng/ml of TGF-β1 for 24 h. **F.** HepG2 cells co-transfected with SBE-luciferase reporter plasmid and either pcDNA3 or Smad3^EPSM^ plasmid together with pCMV or NOX4 plasmid and then stimulated with 5 ng/ml of TGF-β1 for 24 h. Upper panel show the immunoblotting result of siRNA and plasmid transfection. Lower panel show the measured luciferase activity. **G.** HepG2 cells co-transfected with SBE-luciferase reporter plasmid together with pcDNA3 or Smad3^EPSM^plasmid were incubated with or without 100 μM of H_2_O_2_ for 30 min and then stimulated with 5 ng/ml of TGF-β1 for 24 h. Luciferase activities were normalized on the basis of β-galactosidase expression to adjust for variation in transfection efficiency. All quantitative data are shown as the mean ± SD of three independent experiments. **p < 0.01.

NADPH oxidase (NOX) family proteins play important roles in growth factor-induced cell proliferation [31–33] and have recently been recognized as a key component of intracellular signaling triggered by 5-LO metabolites and their receptors [34–35]. In MCF10A cells, LTB_4_ treatment caused a marked increase in ROS production and this increase was significantly abolished by BLT1 siRNA knockdown (Supplementary Figure S2A). In agreement with this result, intracellular ROS levels were much higher in MCF10A-BLT1 cells compared with that of control MCF10A-pcDNA3 cells, and NOX inhibition by treatment with diphenyleneiodonium (DPI), an inhibitor of flavoproteins, significantly diminished the ROS generation in MCF10A-BLT1 cells (Supplementary Figure S2B). Semiquantitative RT-PCR analysis showed that MCF10A-BLT1 cells caused an increase in NOX1 and NOX4 expression levels (Supplementary Figure S2C), suggesting that the LTB_4_/BLT1/NOX axis plays a role in generating intracellular ROS. We thus investigated whether ROS generated by NOX mediate the effect of LTB_4_ signaling on pSmad3L and TGF-β1-induced cell cycle arrest. In Mv1Lu cells, NOX inhibition by Rac1 inhibitor or DPI treatment markedly diminishes LTB_4_-induced Smad3 phosphorylation at Thr179 or Ser208 (Figure 4A). Similarly, depletion of NOX4 by RNAi knockdown clearly diminished the amount of Thr179-, or Ser208-phosphorylated Smad3 in Mv1Lu-BLT1 cells (Figure 4B). Conversely, lentivirus-mediated NOX4 overexpression in MCF10A cells markedly increased the amounts of Smad3 phosphorylated at Thr179 or Ser208 compared with vector control infected cells (Figure 4C). In parallel with this result, treatment of MCF10A cells with H_2_O_2_ resulted in a concentration-dependent increase in the amount of Smad3 phosphorylated at Thr179 or Ser208 (Supplementary Figure S3), indicating an involvement of NOX4 in the induction of pSmad3L by LTB_4_/BLT1 axis. We further assessed the role of NOX4 in LTB_4_-mediated inhibition of TGF-β1-induced Smad3 transcriptional activity. As shown in Figure 4D and 4E, the LTB_4_-, or BLT1-mediated suppression of TGF-β1-induced SBE_4_-Luc reporter gene expression was significantly attenuated by transfection with NOX4 siRNA. Conversely, ectopic expression of NOX4 or H_2_O_2_ treatment drastically reduced TGF-β1-induced SBE_4_-Luc reporter gene expression, but the reduction was largely rescued by cotransfection with Smad3^EPSM^ (Figure 4F and 4G), suggesting that ROS derived by NOX4 function downstream of BLT1 to mediate the suppression of TGF-β1-induced Smad3 transcriptional activity by LTB_4_.

**Figure 5:**
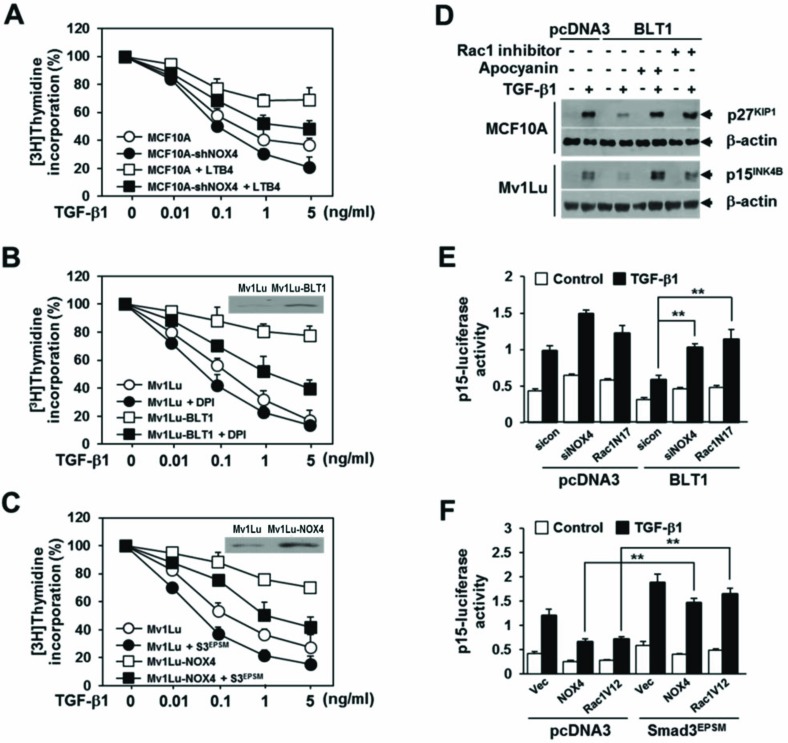
NOX is required for the suppression of TGF-β1-induced growth inhibition by LTB_4_/BLT1 axis **A.** MCF10A cells infected with mock or shNOX4 lentiviruses were incubated with Et-OH (vehicle) or 100 nM of LTB_4_ for 30 min and then stimulated with TGF-β1 at the indicated concentrations for 24 h. **B.** Stable Mv1Lu-pcDNA3 and Mv1Lu-BLT1 cell lines were pretreated with DMSO or 2.5 μM of DPI for 30 min and then stimulated with TGF-β1 at the indicated concentrations for 24 h. Immunoblotting data in the panel show the expression of BLT1. **C.** Mv1Lu cells infected with mock, shNOX4, or both shNOX4 and Smad3^EPSM^ lentiviruses were stimulated with TGF-β1 at the indicated concentrations for 24 h. Immunoblotting data in the panel show the expression of NOX4. Effect of TGF-β1 on cell proliferation was examined using the [^3^H]thymidine incorporation assay. Data are the average of triplicates of three independent experiments and are expressed as percentage of growth (thymidine incorporation relative to control experiment). **D.** MCF10A and Mv1Lu cell lines that stably express pcDNA3 or BLT1 were treated with DMSO, 5 μM of Rac1 inhibitor, or 20 μM of apocyanin for 30 min and then stimulated with 5 ng/ml of TGF-β1 for 24 h. Whole cell lysates were analyzed by immunoblot with a specific antibody against p27^KIP1^ and p15^INK4B^, respectively. β-actin levels were monitored as a control. **E.** HepG2 cells were cotransfected with p15^INK4B^-luciferase reporter plasmid and either pcDNA3 or BLT1 plasmid together with control (scrambled, Scr) or NOX4 siRNAs, or Rac1N17 plasmid and then stimulated with 5 ng/ml of TGF-β1 for 24 h. **F.** HepG2 cells were cotransfected with p15^INK4B^-luciferase reporter plasmid and either pcDNA3 or Smad3^EPSM^ plasmid together with pCMV, NOX4, or Rac1V12 plasmid and then stimulated with 5 ng/ml of TGF-β1 for 24 h. Luciferase activities were normalized on the basis of β-galactosidase expression to adjust for variation in transfection efficiency. All quantitative data are shown as the mean ± SD of three independent experiments. **p < 0.01.

We next investigated whether NOX4 is necessary for the suppression of TGF-β1-induced growth inhibition by LTB_4_/BLT1 signaling axis. Results showed that in MCF10A and Mv1Lu cells, inhibition by LTB_4_ treatment (Figure 5A) or BLT1 overexpression (Figure 5B) of TGF-β1-induced suppression of [^3^H]thymidine incorporation was significantly attenuated by targeted depletion of NOX4 (Figure 5A) or treatment with DPI (Figure 5B). In addition, ectopic expression of NOX4 in Mv1Lu cells strongly inhibited the antiproliferative effect of TGF-β1 on [^3^H]thymidine incorporation, but the inhibition was significantly abolished by co-expression of Smad3^EPSM^ (Figure 5C). Immunoblot analysis revealed that the BLT1-mediated suppression of TGF-β1-induced p15^INK4B^ and p27^KIP1^ expression was completely rescued by Rac1 inhibitor as well as by apocyanin, a specific inhibitor of NOX (Figure 5D). RNAi-mediated depletion of NOX4 or ectopic expression of Rac1N17, a dominant negative form of Rac1, also greatly diminished the inhibitory effect of BLT1 on TGF-β1-induced p15^INK4B^-Luc reporter gene expression (Figure 5E). In contrast, ectopic expression of NOX4 or Rac1V12, a constitutively active form of Rac1, effectively suppressed the reporter gene activity, but the suppression was markedly rescued by Smad3^EPSM^ (Figure 5F). Collectively, these results strongly suggest that NOX plays a pivotal role in the negative regulation of TGF-β1-induced growth inhibition by LTB_4_/BLT1 signaling axis.

### The EGFR-PI3K-ERK1/2-pSmad3L-linked cascade lies downstream of NOX in LTB_4_/BLT1 signal axis

**Figure 6:**
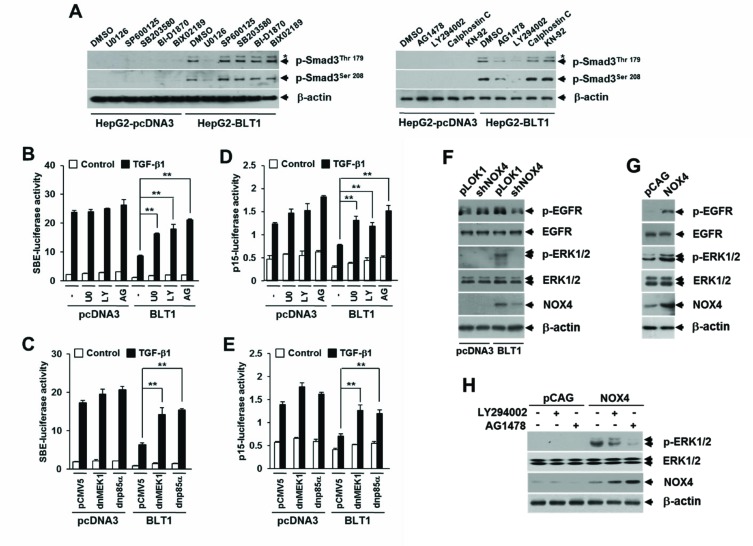
BLT1-NOX-ROS-EGFR-PI3K-ERK1/2-linked cascade constitutes a key LTB_4_ signaling pathway leading to Smad3 linker region phosphorylation **A.** Stable HepG2-pcDNA3 and HepG2-BLT1 cell lines were incubated with DMSO, 5 μM of U0126, 15 μM of SP600125, 15 μM of SB203580, 10 μM of BI-D1870, 10 μM of BIX-02189, 5 μM of AG1478, 20 μM of LY294002, 50 ng/ml of Calphostic C, or 20 μM of KN-92 for 12 h and subjected to immunoblot analysis with specific phosphopeptide antibodies against the phosphorylated Thr179 and Ser208 in the Smad3 linker region. Asterisk represents a phosphor-Smad2 (Thr220). HepG2 cells co-transfected with SBE-luciferase reporter plasmid **B.** or p15^INK4B^-luciferase reporter plasmid **D.** together with pcDNA3 or BLT1 plasmid were treated with DMSO, 5 μM of U0126 (U0), 20 μM of LY294002 (LY), or 5μM of AG1478 (AG) for 30 min and then stimulated with 5 ng/ml of TGF-β1 for 24 h. HepG2 cells were co-transfected with SBE-luciferase reporter plasmid **C.** or p15^INK4B^-luciferase reporter plasmid **E.** and either pcDNA3 or BLT1 plasmid together with pCMV5 or dominant negative (dn) forms of MEK1 (dnMEK1) or p85α (dnp85α) and then stimulated with 5 ng/ml of TGF-β1 for 24 h. Luciferase activities were normalized on the basis of β-galactosidase expression to adjust for variation in transfection efficiency. All quantitative data are shown as the mean ± SD of three independent experiments. **p < 0.01. **F.** Stable HepG2-pcDNA3 and HepG2-BLT1 cell lines were infected for 36 h with pLKO1 (control) or shNOX4 lentivirus. **G.** HepG2 cells were infected for 36 h with pCAG (control) or NOX4 lentivirus. **H.** MCF10A cells infected with pCAG (control) or NOX4 lentiviruse were treated with DMSO, 20 μM of LY294002 or 5 μM of AG1478 for 12 h. Whole cell lysates were analyzed by immunoblot with a specific antibody against EGFR, phospho-EGFR, ERK1/2, phospho-ERK1/2, or NOX4, respectively. β-actin levels were monitored as a control.

The Smad3 linker domain undergoes regulatory phosphorylation by several intracellular signaling kinases [21, 22] To identify a signaling kinase involved in the LTB_4_-mediated pSmad3L,we tested the effects of inhibitors specific to several candidate kinases, including AG1478 (an epidermal growth factor receptor inhibitor), Calphostin C (a general protein kinase C inhibitor), KN-92 (a calcium-calmodulin-dependent protein kinase II inhibitor), LY294002 (a phosphatidylinositol 3-kinase inhibitor), BI-D1870 (a p90 ribosomal S6 kinase inhibitor), BIX02189 (an extracellular signal-regulated kinase 5 inhibitor), U0126 (a mitogen-activated protein-ERK kinase (MEK) 1/2 inhibitor), SP600125 (a c-Jun NH_2_-terminal kinase inhibitor), and SB203580 (a p38 kinase inhibitor). Immunoblot analysis revealed that the increased amounts of Smad3 phosphorylated at Thr179 or Ser208 in Mv1Lu-BLT1 cells were markedly diminished by treatment with AG1478, LY294002, or U0126, whereas similar concentrations of other inhibitors had no significant effects (Figure 6A). LTB4-induced pSmad3L at Thr179 or Ser208 was also abrogated by treatment with U0126 in a concentration-dependent manner (Supplementary Figure S4). These results indicate an involvement of EGFR, PI3-kinase, and ERK1/2 in LTB_4_ signaling pathway leading to the phosphorylation of pSmad3L. We next examined whether activation of these candidate kinases is responsible for the BLT1-mediated suppression of cytostatic TGF-β1 responses. We found that the BLT1-mediated suppression of TGF-β1-induced SBE_4_-Luc (Figure 6B and 6C) and p15^INK4B^-Luc (Figure 6D and 6E) reporter gene expression was significantly attenuated by treatment with U0126, LY294002, or AG1478, or by co-transfection with a plasmid expressing dominant negative form of MEK1 or p85α. Furthermore, we established that an EGFR-PI3-kinase-ERK1/2-linked cascade lies downstream of NOX4 in LTB_4_/BLT1 signaling (Figure 6F, 6G, and 6H). Taken together, these results suggest that the NOX-ROS-EGFR-PI3K-ERK1/2-pSmad3L-linked cascade constitutes a fundamental intracellular signaling pathway that mediates LTB_4_ signal from BLT1 receptor, and activation of this pathway is responsible for the suppression of antiproliferative TGF-β1 signal by LTB_4_.

### Enhanced pSmad3L through BLT1-Rac1/NOX4-EGFR-ERK1/2 linked signaling contributes to the resistance of MDA-MB231 breast cancer cells to the TGF-β1 growth-inhibitory response *in vitro* and *in vivo*

**Figure 7:**
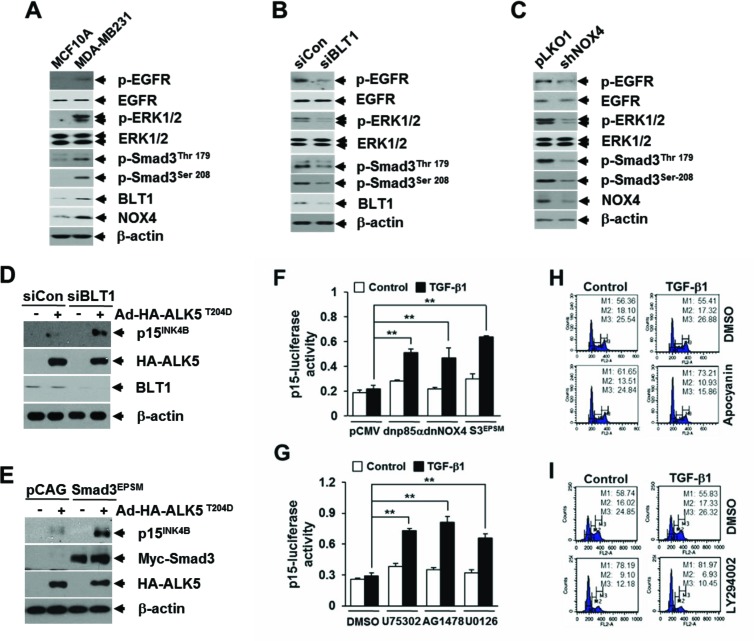
BLT1 signaling to Smad3 linker region phosphorylation contributes to the resistance to TGF-β1 growth inhibition of MDA-MB231 breast cancer cells **A.** MCF10A and MDA-MB231 cells, **B.** MDA-MB231 cells transfected with BLT1 (siBLT1) or control (siCon), and **C.** MDA-MB231 cells infected with mock or shNOX4 lentiviruses were harvested and subjected to immunoblot analysis using antibodies against phospho-Smad3 (Thr179), phospho-Smad3 (Ser208), ERK1/2, phospho-ERK1/2, EGFR, phospho-EGFR, NOX4 and BLT1. B-actin levels were monitored as a control. Asterisk represents a phospho-Smad2 (Thr220). The β-actin level was monitored as controls. **D.** MDA-MB231 cells transfected with control siRNA or BLT1 siRNA were infected with mock or HA-ALK5^T204D^ adenoviruses and subjected to immunoblot analysis using antibodies against BLT1, p15^INK4B^ and HA. **E.** MDA-MB231 cells were co-infected with mock or Smad3^EPSM^ lentiviruses together with ALK5^T204D^ adenoviruses and subjected to immunoblot analysis using antibodies against p15^INK4B^, Myc, HA, and β-actin levels were monitored as controls. **F.** MDA-MB231 cells were co-transfected with p15^INK4B^-luciferase reporter plasmid together with pCMV5, dominant negative (dn) forms of p85α (dnp85α) or NOX4 (dnNOX4), or Smad3^EPSM^ and then stimulated with 5 ng/ml of TGF-β1 for 24 h. **G.** MDA-MB231 cells transfected with p15^INK4B^-luciferase reporter plasmid were treated with DMSO, 10 μM of U75302, 5 μM of AG1478, or 5 μM of U0126 for 30 min and then stimulated with 5 ng/ml of TGF-β1 for 24 h. Luciferase activities were normalized on the basis of β-galactosidase expression to adjust for variation in transfection efficiency. All quantitative data are shown as the mean ± SD of three independent experiments. **p < 0.01. MDA-MB231 cells pretreated with 20 μM of apocyanin **H.** or 20 μM of LY294002 **I.** were incubated with 5 ng/ml of TGF-β1 for 24 h in the absence or presence of 100 nM of LTB_4_. Cells were then stained with propodium iodide and subjected to FACS analysis. The percentage of cells in G1 was designated as M1, S as M2, and G2/M as M3.

Loss of response to growth inhibition induced by TGF-β1 is closely linked to the occurrence of cancer. We thus evaluated the significance of aforementioned LTB_4_ signaling pathway in TGF-β1 resistance of cancer cells. To do this, we chose MDA-MB231 human breast cancer cells that were refractory to TGF-β1-induced growth inhibition (Supplementary Figures S5A and S5B). FACS and immunoblot analysis revealed that abundances of intracellular ROS (Supplementary Figure S6), BLT1 and NOX4 proteins, and phosphorylated forms of Smad3 (Thr179 and Ser208), ERK1/2, and EGFR proteins (Figure 7A) were markedly greater in TGF-β1-resistant MDA-MB231 breast cancer cells compared with TGF-β1-responsive non-malignant MCF10A cells. Depletion of BLT1 (Figure 7B) or NOX4 (Figure 7C) by siRNA-mediated knockdown in MDA-MB231 cells resulted in a marked reduction of the amount of phosphor-Smad3 (Thr179 and Ser208), phosphor-ERK1/2, and phosphor-EGFR. Similar effects were observed in MDA-MB231 cells treated with U75302 (Supplementary Figure S7A) or apocyanin (Supplementary Figure S7B). We then examined whether the BLT1-linked signaling cascade significantly contributes to the resistance of MDA-MB231 cells to TGF-β1-induced growth inhibition. RNAi-mediated silencing of BLT1 (Figure 7D) or ectopic expression of Smad3^EPSM^ (Figure 7E) markedly abolished the resistance of MDA-MB231 cells to TβRI(T204D)-induced p15^INK4B^ expression. TGF-β1-induced p15^INK4B^-Luc reporter gene expression was also significantly enhanced by co-transfection with a plasmid expressing Smad3^EPSM^ or dominant-negative forms of p85α or NOX4 (Figure 7F), or by treatment with U75302, AG1478, or U0126 (Figure 7G). Furthermore, FACS analysis revealed that co-treatment of TGF-β1 with apocyanin (Figure 7H) or LY294002 (Figure 7I) substantially increased the accumulation of MDA-MB231 cells in G_1_ phase compared with TGF-β1 treatment alone.

**Figure 8:**
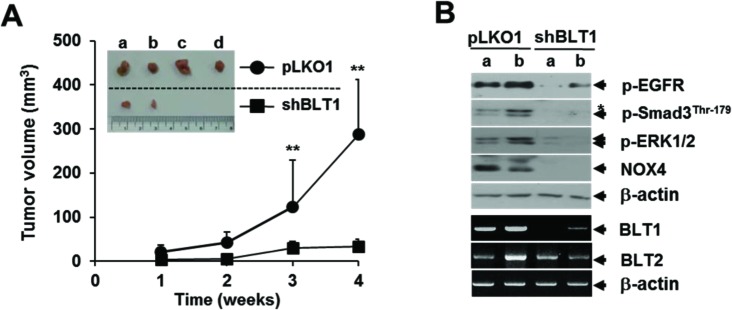
Lentivirus-mediated knockdown of BLT1 by shRNA inhibits the xenograft tumor growth of MDA-MB231 cancer cells in nude mice **A.** Suspensions of 1 × 10^7^ MDA-MB231-pLKO1 or MDA-MB231-shBLT1 stable cells in 0.2 ml of PBS were injected subcutaneously into the flanks of Balb/c nude mice (n=5). The inset shows the representative tumors at 5 weeks after injection. Tumor volumes were measured weekly using a microcaliper for 4 weeks. Data are representative of results obtained with five mice per group. **p < 0.01. **B.** Proteins and mRNAs extracted from the xenograft tumors were subjected to both immunoblot analysis for phospho-EGFR, phospho-Smad3 (Thr179), phospho-ERK1/2, and NOX4 levels and semiquantitative RT-PCR analysis for BLT1 and BLT2 mRNA levels. β-actin and GAPDH levels were monitored as a loading control for tumor extracts. Asterisk represents a phospho-Smad2 (Thr220). Number of animals per group = 5.

**Figure 9:**
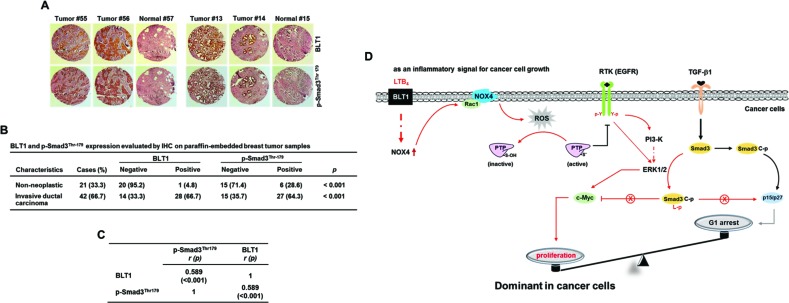
pSmad3L and BLT1 are positively correlated in human breast tumor tissues **A.** Expression levels of pSmad3L and BLT1 in normal adjacent tissues and breast tumors were examined using an immunohistochemical staining assay of breast cancer patient samples. **B.** Statistical analysis of the correlation between the protein levels of BLT1 and pSmad3L in the breast cancer tissue array. **C.** The expression correlation between BLT1 and p-Smad3Thr179 in the human breast cancer tissues was significant as determined by Kendall tau rank correlation test. *r*: Kendall correlation coefficient. **D.** A model illustrating the mechanism underlying promotion of cancer cell proliferation by proinflammatory LTB_4_/BLT1 signal. In cancer cells, LTB_4_-evoked BLT1 activation induces production of ROS by increasing NOX4 association with membrane, resulting in activation of EGFR through protein tyrosine phosphatase (PTP) oxidation. The activated receptor induces the phosphorylation of Smad3 in the linker region through a EGFR-PI3K-ERK1/2- and EGFR-ERK1/2-linked signaling pathways, which leads to blockade of suppression of c-myc expression as well as induction of cyclin-dependent kinase inhibitors (p15^INK4B^ and p27^KIP1^) in TGF-β signaling necessary for growth inhibition. The proposed mechanism suggests that the proinflammatory LTB_4_/BLT1 signal contributes to promotion of cancer cell proliferation by tipping growth balance to cell cycle progression through inhibition of cytostatic TGF-β1 signaling.

To evaluate our *in vitro* findings on MDA-MB231 cell growth *in vivo*, we xenografted the following cell types subcutaneously in the right flank of athymic nude mice: MDA-MB231-shBLT1 cells that stably expressing shRNA against human BLT1 and MDA-MB231-pLKO1 cells that stably expressing control vector. At a postmortem examination conducted after 28 days, we observed that tumors derived from MDA-MB231-shBLT1 cells grew at a much slower rate than those from control MDA-MB231-pLKO1 cells (Figure 8A). In addition, the MDA-MB231-shBLT1 xenograft tumors showed the reduced levels of BLT1 and NOX4 proteins and EGFR, Smad3^Thr179^, and ERK1/2 phosphorylation compared with those of MDA-MB231-pLKO1 tumors (Figure 8B). Furthermore, immunohistochemistry analysis on human breast tumor tissue micro-array revealed that the protein levels of BLT1 and p-Smad3^Thr179^ were greater in tumor tissues compared with those in normal tissues (Figure 9A and 9B). Kendall tau rank correlation test showed the positive correlation between BLT1 and p-Smad3Thr179 expression in the human breast cancer tissues (Figure 9C). Collectively, these data suggest that enhanced pSmad3L through BLT1-NOX-ROS-EGFR-PI3K-ERK1/2-linked signaling cascade as a critical mechanism for the resistance of breast cancer cells to TGF-β1-induced growth inhibition.

## DISCUSSION

Pro-inflammatory mediators released from tumor and stromal cells in tumor microenvironment play critical roles in fueling tumor development. Recently, growing evidences have implicated LTB_4_, a proinflammatory eicosanoid, in a number of processes linked to cancer progression, but limited information is available regarding molecular mechanisms for its action in cancer. This study demonstrated a new role for LTB_4_ as a negative regulator at a key point of TGF-β1 signaling necessary for growth inhibition, and revealed that blockade of BLT1 expression sensitizes breast cancer cells to TGF-β1-induced growth inhibition both *in vitro* and *in vivo*. Results mechanistically showed increased pSmad3L through BLT1/NOX4/ROS/EGFR/PI3-K/ERK1/2-linked signaling cascade is crucial for LTB_4_-mediated inhibition of TGF-β1-induced cell cycle arrest at the G1 phase. This is the first report to show inflammatory LTB_4_/BLT1 signal in control of cancer cell proliferation through the inhibition of anti-proliferative TGF-β1 activity. These findings thus provide a mechanism that allows connection between inflammatory signal and tumor growth.

Most of LTB_4_ signaling research in relation to cancer progression has been focused on the role of BLT2. The study by Kim H *et al*. showed that oncogenic Ras promotes TGF-β1-induced epithelial-to-mesenchymal transition via a BLT2-linked signaling pathway in mammary epithelial cells [36]. BLT2 has also been shown to mediate lipopolysaccharide-potentiated invasiveness and metastasis of breast cancer cells [37]. In this study, we observed an inhibitory effects of BLT2 in TGF-β1-induced cell cycle arrest and p15^INK4B^ expression (data not shown). However, since BLT2 was characterized to function as a low affinity receptor, with broader ligand specificity for various eicosanoids, including HETEs and HPETEs [38], our study has focused on BLT1 with ligand specificity for LTB_4_ only [39]. In mammary epithelial cells, LTB_4_ strongly inhibited TGF-β1-induced cell cycle arrest at the G1 phase and this effect was dependent on BLT1 (Figure 1). Notably, FACS analysis showed that LTB_4_ by itself does not significantly stimulate cell cycle progression from G_1_ to S phase, suggesting that enhanced LTB_4_-BLT1 axis in cancer cells may facilitate proliferation by tipping growth balance to cell cycle progression through inhibition of TGF-β1 growth-inhibitory response.

Previous studies have established that Smad3 has a key function in mediating the anti-proliferative TGF-β1 response [19, 20]. However, recent growing evidences suggested that Smad3-phosphoisoforms have different roles in TGF-β signaling; C-terminally phosphorylated Smad3 transmits cytostatic TGF-β signal, whereas pSmad3L is induced by oncogenes or mitogenic signals and contributes to a shift from tumor suppressive to oncogenic activity [40] In addition, increased pSmad3L level has been frequently observed in several types of pathological conditions such as chronic inflammation, fibrosis, and cancer [41–43] In line with these reports, our data showed that LTB_4_/BLT1 inflammatory signal axis impairs TGF-β1-induced Smad3 transcriptional activity by inducing pSmad3L without affecting its canonical activation processes. Mutation of all four sites in the Smad3 linker region can largely rescue the inhibition by LTB_4_/BLT1 axis of anti-proliferative TGF-β1 response. In MDA-MB231 xenografts, knockdown of BLT1 significantly reduced the level of pSmad3L at Thr179 concomitant with tumor growth inhibition. Importantly, immunohistochemical staining of human breast tumors has provided support for the relevance of BLT1 overexpression and increased pSmad3L. Therefore, our study suggests that Smad3 linker region can be an important target of LTB_4_ inflammatory signal leading to cancer cell proliferation.

A recent study by O'Leay et al. showed that TLR/NOX1 redox signaling axis accelerate colon cancer cell adhesion, thus increasing metastatic potential of the colon cancer cells [44] Similarly, NOX1-derived ROS generation had impact on TLR4 signaling to enhance tumor metastasis of non-small cell lung cancer (NSCLC) [45]. NOX-derived ROS generation is also essential for activation of signaling pathways involved in cell proliferation through tyrosine phosphatase oxidation and subsequently sustained tyrosine kinase receptor phosphorylation [46]. Li *et al*. reported that reciprocal activation between IL-6/STAT3 and NOX4/Akt signaling promotes proliferation of NSCLC [47]. These evidences indicate an important role of NOX enzyme in the pathogenesis of inflammation-associated tumor development. Nuclear factor-kappaB (NF-κB) has been shown as a major transcription factor that regulates NOX4 gene expression [48, 49], and LTB_4_ activates NF-κB pathway [50]. Consistent with these findings, our data showed that BLT1 increased NF-κB-dependent reporter gene activity, and BLT1-induced NOX4 expression was strongly inhibited by NF-κB inhibitor, pyrrolidine dithiocarbamate hydrochloride (Supplementary Figure S8). We demonstrated in this study that enhanced NOX1 and NOX4 expression and ROS production triggered by LTB_4_/BLT1 signal axis increases the pSmad3L, contributing to the resistance to the TGF-β1 growth-inhibitory effects in cancer cells. Furthermore, our study identified the EGFR-PI3K-ERK1/2-linked cascade as a major signaling route connecting BLT1/NOX-derived ROS generation and pSmad3L. These findings suggest a novel functionality of NOX in the context of inhibition of Smad3 tumor suppressive function through linker phosphorylation level. Inhibition of NOX can contribute to decreased levels of pSmad3L, and hence help restore TGF-β1 growth-inhibitory response in cancer cells.

In summary, we showed that LTB_4_ can target Smad3 linker region through a ‘BLT1-NOX-ROS-EGFR-PI3K-ERK1/2’ signaling cascade. This contributes to the resistant to the TGF-β1 growth-inhibitory effects, thus increased proliferation of highly aggressive breast cancer cells (Figure 9D). Therefore, the elucidation of this mechanism provides an important insight into how proinflammatory LTB_4_ promotes the proliferation of breast cancer cells.

## MATERIALS AND METHODS

### Reagents and antibodies

LTB_4_ and U75302 were purchased from Cayman Chemicals (Ann Arber, MI). The recombinant TGF-β1 was purchased from R&D systems (Minneapolis, MN). AG1478, LY294002, KN-92, Calphostin C, U0126, PD169316, SP600125, BI-D1870, BIX02189, DPI, apocyanin, and Rac1 were obtained from Calbiochem (La Jolla, CA). H_2_DCFDA was purchased from Molecular Probes (Eugene, OR). Human BLT1-specific (5′-UACUCCCACCACAAAGCUGUUGCC-3′) siRNA was obtained from Bioneer (Daejeon, Korea). Myc, p27^KIP1^, and p15^INK4B^ antibodies and control siRNA were from Santa Cruz Biotechnology (Santa Cruz, CA). Smad3, phospho-Smad3, ERK1/2, phospho-ERK1/2, EGFR and phosphor-EGFR antibodies were from Cell Signaling Technology (Danvers, MA). BLT1 and NOX4 antibodies were from abcam (Cambridge, MA). β-actin antibody was from Sigma-Aldrich (St. Louis, MO).

### Cell lines

The HepG2 human hepatoblastoma cells, MCF10A human mammary epithelial cells, MDA-MB231 human breast cancer cells, and Mv1Lu mink lung epithelial cells were purchased from the American Type Culture Collection (Rockville, MD). Eph4 mouse mammary epithelial cells were provided by the late Anita B. Roberts (National Cancer Institute, USA). The HepG2-BLT1 and Mv1Lu-BLT1 stable clones that expressing BLT1 were prepared by transfection with pCDNA3-BLT1 encoding Flag-tagged human BLT1, followed by selection with 0.5 mg/ml of G418 (Invitrogen, Carlsbad) as described [24]. The MDA-MB231-shBLT1 clones were obtained by infection with lentivirus containing shRNA BLT1, followed by selection with 0.2 mg/ml of puromycine (Invitrogen, Carlsbad) for 15 days.

### Plasmid constructs

To silence the expression of NOX4 and BLT1, the following oligonucleotides were cloned into pLKO.1-TRC shRNA vector; for NOX4 (5′-AGCAAGATACCGAGATGAGGA-3′ corresponding to nucleotides 428-449 downstream of the transcription start site of human NOX4) and BLT1 (5′-GGCAACAGCTTTGTGGTGT-3′ corresponding to nucleotides 102-120 downstream of the transcription start site of human BLT1). These sequences were separated by a 6-nucleotide noncomplementary spacer (CTCGAG) from the reverse complement of the target nucleotide sequence and inserted into pLKO-TRC digested with AgeI and EcoRI; the resulting vectors were designated pLKO-shNOX4 and pLKO-shBLT1. The human BLT1 plasmid was kindly provided by Dr. Takao Shimizu (University of Tokyo, Tokyo, Japan). The NOX4 expression plasmid was a gift from Dr. Yun Soo Bae (Ewha Woman's University, Seoul, Korea).

### Semiquantitative RT-PCR

Total cellular RNA was extracted from cells using the phenol-guanidinium isothiocyanate method [25]. Two microgram of RNA was reverse-transcribed for 1 h at 42°C and amplified by PCR using specific primers for human BLT1 (sense, 5′-TATGTCTGCGGAGTCAGCATGTACGC-3′; antisense, 5′-CCTGTAGCCGACGCCCTATGTCCG-3′), human NOX4 (sense, 5′-GTTCTTAACCTCAACTGCAGCC-3′; antisense, 5′-GCATATGTAGAGGCTGTGATC-3′), and GAPDH (sense, 5′-CTGCACCACCAACTGCTTAGC-3′; antisense, 5′-CTTCACCACCTTCTTGATGTC-3′); internal control).

### Electrophoretic mobility shift assay

Nuclear extracts were prepared as previously described [26]. Double-stranded oligonucleotides containing Smad-binding element (5′-AGTATGTCTAGACTGA-3′) were labeled with [γ-^32^ATP] and T4 polynucleotide kinase. DNA-binding assay (20 μl final volume) was carried out for 30 min at 4°C, with 10 μg of nuclear extracts, 30, 000 cpm of ^32^P-labelled probes, and 1 μg of poly(dI-dC) [poly(deoxyinosinic-deoxycytidic acid)] in 10 mM Tris-Cl, pH 7.5, 75 mM KCl, 1 mM DTT, and 5% glycerol. For supershift assay, the extracts were incubated with 0.5 μg of Smad3 antibody for 1 h at 4°C and were loaded onto 4% nondenaturating polyacrylamide gel. After electrophoresis, gels were dried, exposed to imaging plates, and analyzed.

### Cell proliferation assay

DNA synthesis in cells was measured by [^3^H]-thymidine incorporation. Cells were incubated with various concentrations of TGF-β1 for 20 h and were pulsed with 0.25 μCi of [methyl-^3^H]-thymidine (40 to 60 Ci/mmol, NEN Life Science Products, Inc., Boston, MA) for 2 h at 37°C. At the end of this period, cells were rinsed in PBS and fixed with methanol-acetic acid (3:1, v/v) for 1 h. Cells were dissolved in NaOH (0.5 M) and the amount of [^3^H]-thymidine incorporated was measured using liquid scintillation counting.

### Xenograft studies

Animal experimental procedures were approved by the Institutional Animal Care and Use Committee of Kangwon National University. Female Balb/c nude mice (4 weeks, 16 ~ 20 g) were purchased from Orient Bio (Seongnam, Korea) and allowed to acclimatize for 1 week. Suspensions of 1 × 10^7^ MDA-MB231-pLKO1 or MDA-MB231-shBLT1 stable cells in 0.2 ml of PBS were injected subcutaneously into the flanks of mice (n=5 per group). Tumor volumes were measured weekly using a microcaliper for 4 weeks and calculated by utilizing the following formula: *TV* (mm^3^)=*L* x (*W*)^2^/2, where *TV*=tumor volume, *L*=length and *W*=with. On day 28, mice were euthanized according to the institutional guidelines for the care and use of laboratory animals, and tumors were excised. Proteins and mRNAs extracted from the xenografted tumors were subjected to immunoblot and semiquantitative RT-PCR analysis, respectively.

### Immunohistochemical analysis

Immunohistochemistry for phospho-Smad3 at Thr^179^ and BLT1 was performed using previously described method [27]. Rabbit monoclonal antibody against BLT1 (ab131041, abcam, Cambridge, MA) and rabbit polyclonal antibody against phosphor-Smad3 at Thr^179^ (ab74062, abcam, Cambridge, MA) was used with 1:50 dilutions. The human breast tissue array consisting of 42 paired human breast cancer tissues with corresponding normal tissues was from AccuMax array (A312 (II) breast cancer, Petagen Inc., Seoul, Korea). Negative or positive immunostaining from all stained slides was counted and recorded by two independent investigators under a Nikon ECLIPSE 80i Light Microscope and representative photographs were taken.

### Additional methods

Transfection, viral infection, luciferase assay, immunofluorescence staining, flow cytometry analysis, immunoblotting, and measurement of intracellular ROS were as previously described [28].

### Statistical analysis

Statistical analyses were performed using SigmaPlot 2001 (Systat Software, Inc, Richmond, CA). Statistical significance was assessed by comparing the means values (±SD) using a Student's *t*-test for paired data. We determined the significance of differences in the human tissue data using Kendall tau rank correlation test. *P* values less than 0.05 were considered statistically significant.

## SUPPLEMENTARY MATERIAL FIGURES


