# Supplementary material for: The proinflammatory LTB4/BLT1 signal axis confers resistance to TGF-β1-induced growth inhibition by targeting Smad3 linker region
Source: Oncotarget. 2015 Oct 19;6(39):41650–66. doi: 10.18632/oncotarget.6146 (PMC4747179; doi:10.18632/oncotarget.6146)
Supplement: Supplementary file 1 [file oncotarget-06-41650-s001.pdf]

# The proinflammatory $LTB_4$/BLT1 signal axis confers resistance to TGF-β1-induced growth inhibition by targeting Smad3 linker region

**Supplementary Material**

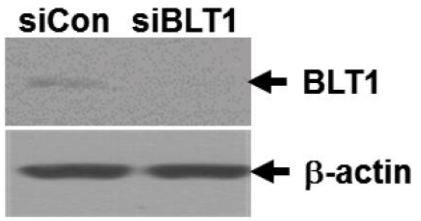


**Supplementary Figure 1: Immunoblot analysis that showing the expression of endogenous BLT1 in MCF10A cells transfected with control siRNA and BLT1 siRNA. β-actin levels were monitored as a control.**

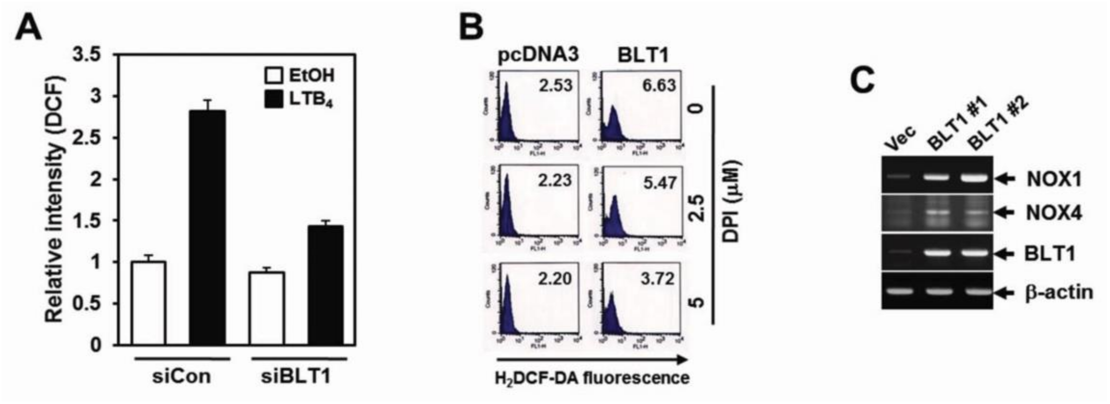


**Supplementary Figure 2: $LTB_4$ increases intracellular ROS production through BLT1-mediated up-regulation of NOX. (A) Inhibition of $LTB_4$-induced ROS production by knock-down of BLT1. (B) Inhibition of BLT1-induced ROS generation by DPI. (C) Increases of NOX1 and NOX4 mRNA expression in MCF10A cells that stably expressing BLT1.**

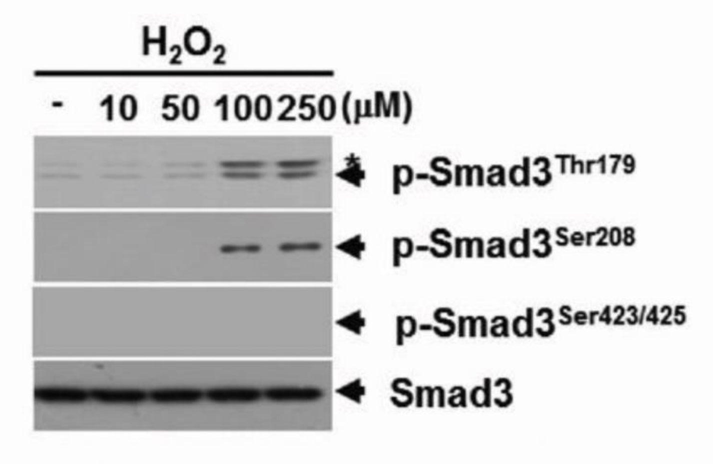


**Supplementary Figure 3: $H_2O_2$ increase phosphorylation of Smad3 at the linker region.**

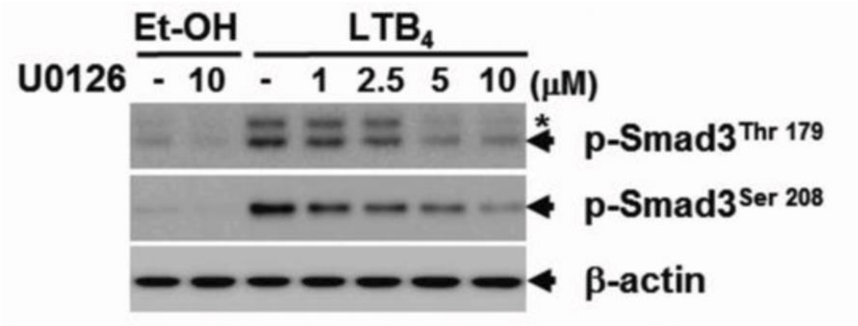


**Supplementary Figure 4: U0126 inhibits $LTB_4$-induced phosphorylation of Smad3 at the linker region.**

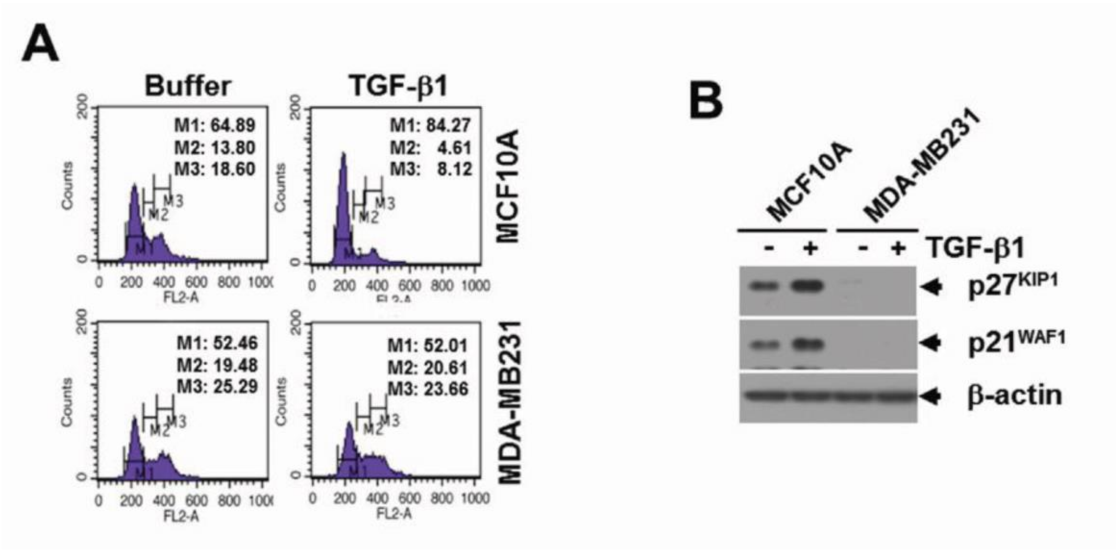


**Supplementary Figure 5: Resistance of MDA-MB231 human breast cancer cells to TGF-β1-induced growth inhibitory response. FACS analysis (A) that showing an arrest in cell cycle progression at $G_1$ and immunoblot analysis (B) that showing the expression of $p27^{KIP1}$ and $p21^{WAF1}$ proteins in MCF10A and MDA-MB231 cells in response to TGF-β1.**

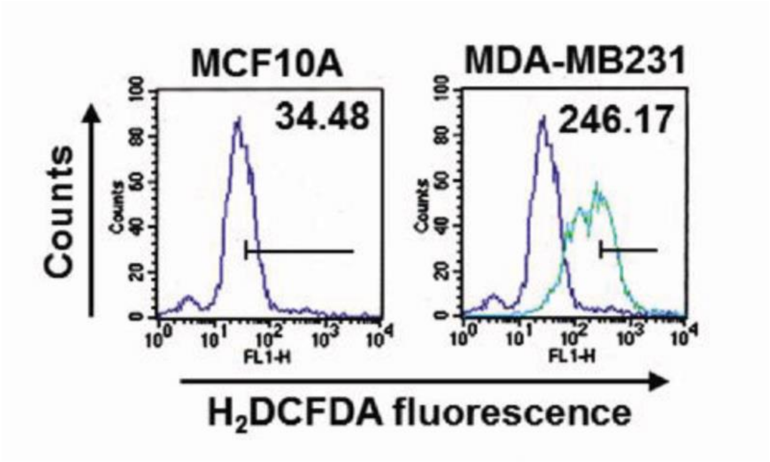


**Supplementary Figure 6: Intracellular ROS levels in MCF10A and MDA-MB231 cells.**
**The intracellular ROS levels were determined by measuring DCFDA fluorescence with a flow cytometer.**

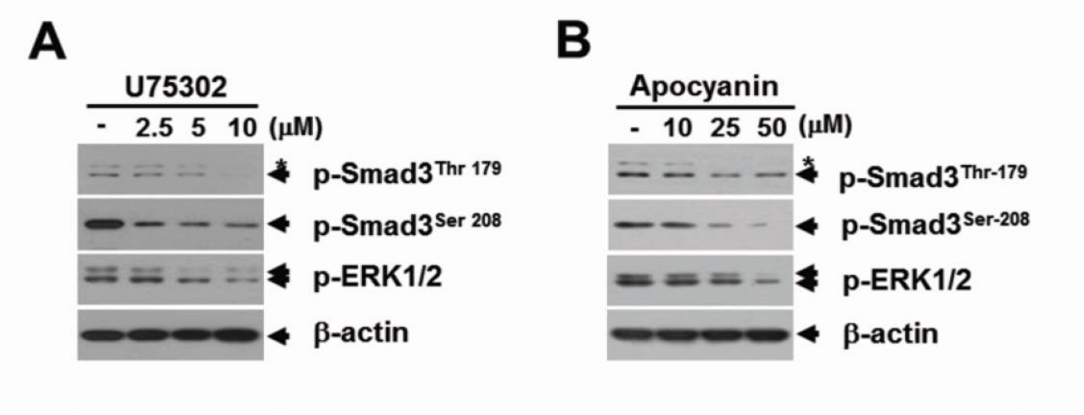


**Supplementary Figure 7: Decreased phosphorylations of Smad3 linker region and ERK1/2 by U75302 (A) or apocyanin (B) in MDA-MB231 cells.**

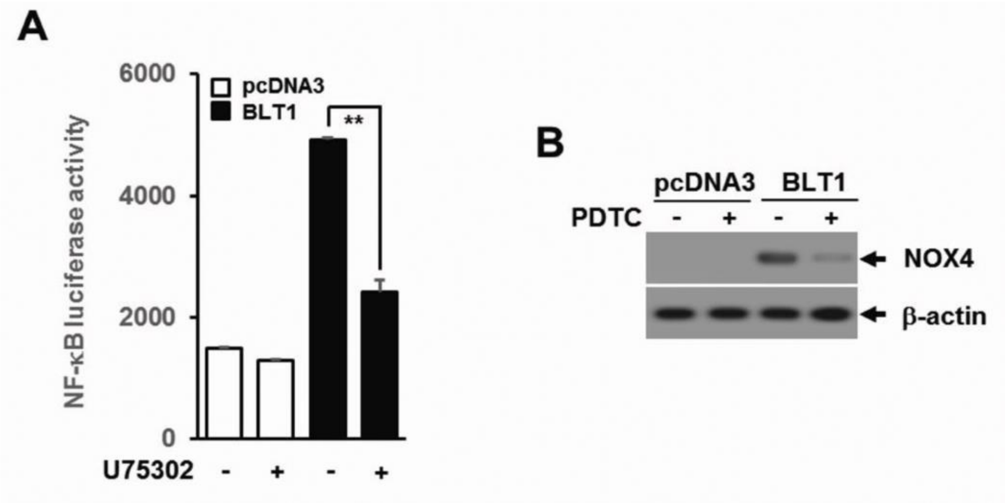


**Supplementary Figure 8: NF-κB mediates BLT1-induced NOX4 expression**
**(A) Inhibition of BLT1-induced NF-κB reporter gene activity by U75302 (10 μM). (B) Inhibition of BLT1-induced NOX4 expression by NF-κB inhibitor PDTC.**
